# Natural Molecular Mechanisms of Plant Hyperaccumulation and Hypertolerance towards Heavy Metals

**DOI:** 10.3390/ijms23169335

**Published:** 2022-08-19

**Authors:** Lidia Skuza, Izabela Szućko-Kociuba, Ewa Filip, Izabela Bożek

**Affiliations:** 1 Institute of Biology, University of Szczecin, 13 Wąska, 71-415 Szczecin, Poland; 2 The Centre for Molecular Biology and Biotechnology, University of Szczecin, 13 Wąska, 71-415 Szczecin, Poland

**Keywords:** hyperaccumulation, hypertolerance, phytoremediation, heavy metals, plant tolerance

## Abstract

The main mechanism of plant tolerance is the avoidance of metal uptake, whereas the main mechanism of hyperaccumulation is the uptake and neutralization of metals through specific plant processes. These include the formation of symbioses with rhizosphere microorganisms, the secretion of substances into the soil and metal immobilization, cell wall modification, changes in the expression of genes encoding heavy metal transporters, heavy metal ion chelation, and sequestration, and regenerative heat-shock protein production. The aim of this work was to review the natural plant mechanisms that contribute towards increased heavy metal accumulation and tolerance, as well as a review of the hyperaccumulator phytoremediation capacity. Phytoremediation is a strategy for purifying heavy-metal-contaminated soils using higher plants species as hyperaccumulators.

## 1. Introduction

Heavy metals are a group of elements with a high density, i.e., above 5 g/cm^3^ [1]. These include metallic elements (Mn, Zn, Cu, Hg, and Cd) and metalloids (Se and As). These elements may be biogenic, i.e., essential for proper plant functioning, since they regulate the processes of photosynthesis, respiration, nitrogen metabolism (Fe, Zn, Cu, and Co, among others), or toxic, that cause diseases and disrupt many plant processes [2,3]. Soil trace metal sources can be classified as natural and anthropogenic. Naturally occurring soil metals result from rock weathering or volcanic eruptions and are less bioavailable compared to sources of anthropogenic origin. Man contributes to high heavy metal soil concentrations through mining, agriculture (fertilization), metallurgy, or fuel combustion and transport [4,5]. Not only do these sources seriously threaten humans, but also animals and plants. Heavy metals can contribute, among other things, to protein synthesis changes and ATP production disorders, which can cause serious pathological changes, including cancer. Soil heavy metals inhibit rhizospheric microorganism development, which decreases their degradation efficiency as well as organic compound transformation. When microorganism growth is inhibited, plant pathogen resistance decreases, as does plant development [6]. Fortunately, certain plants can establish in areas with a high heavy metal content. These plants have characteristics that enable them to survive adverse conditions, including a tolerance to high soil heavy metal concentrations [7]. This emphasizes the importance of understanding the mechanisms by which plants absorb high metal concentrations, as well as their depollution of contaminated soils.

This paper presents a literature review of the natural plant mechanisms which contribute to increased heavy metal accumulation and tolerance, as well as the hyperaccumulator phytoremediation capacity.

## 2. Naturally Occurring Heavy Metal Hyperaccumulators

A hyperaccumulator is a plant which can absorb and accumulate heavy metals in its above-ground sections (especially the leaves) at values exceeding specific metal thresholds [4]. These values are reportedly 10–500 times higher than in other plants, and hyperaccumulators exhibit no phytotoxic symptoms [2,8]. Trace metals can be taken up by the plant at different rates, depending on, among other things, soil pH, soil organic matter content, metal type, and whether other soil ions are present (which can be antagonistic) [3]. The main plant tolerance mechanism is based on metal uptake avoidance, or on uptake and neutralization through specific plant processes [9]. Plants reportedly take up and accumulate large amounts of heavy metals as an allelopathic defense strategy against competing plants; metal accumulation can also serve as a defense against drought or as a defense strategy against herbivores and pathogens [4] (Table 1).

Many studies on hyperaccumulators have confirmed the above-mentioned defensive plant mechanisms. Transcriptomic studies on *Arabidopsis* *halleri* have shown that excessive metal accumulation is associated with an increased expression of more than 30 genes, while in *Noccaea caerulescens*, the Zinc Transporter 1 (ZnT1) increases in expression [14].

All these mechanisms allow plants to adapt to stress factors. However, it is possible that heavy metal ions can be unbound and not transported, e.g., to a vacuole, and thereby remain in metal-sensitive areas [1]. Under such conditions, the plant may form reactive oxygen species (ROS) which cause oxidative stress. Excess ROS can damage and reduce antioxidant pathway activity, while in chloroplasts, excess ROS inhibits photosynthesis [16]. This contributes to apoptosis or defense system activation through gene expression, i.e., antioxidant defense system activation [12,17].

### 2.1. Classification and Occurrence of Hyperaccumulators

Metallophytes, found in heavy-metal-contaminated areas, occur in 34 unrelated flowering plant families. Most metallophytes belong to the Brassicaceae (25%), Asteraceae, Cayrophyllaceae, Plumbaginaceae, Cyperaceae, Violaceae, Poaceae, Fabaceae, and Euphorbiaceae [2,4,18]. About 450 flowering plant species are known hyperaccumulators, which represents about 0.2% of all known species, although this number is still growing. However, some species may be removed from the hyperaccumulator list, which happens when they are classified only based on field samples, and where the trait has not been experimentally confirmed under controlled conditions [8]. To avoid such situations, it is necessary to thoroughly analyze and evaluate a plant as a hyperaccumulator.

A plant may accumulate one (most frequently) or several metals [8]. Most hyperaccumulators are flowering dicotyledonous and herbaceous plants. Most hyperaccumulators (90%), such as *Alyssum* *discolour*, *Alyssum inflatum*, *Minuartia baldaccii*, and *Viola* *dukadjinica*, are endemic plants found on serpentine soils (rich in Ni, Co, Cr, and Mn) [19,20]. However, hyperaccumulators can also be found on calamine soils (rich in Pb, Zn, and Cd). For example, *Armeria elongata*, *Silene vulgaris*, *Biscutella* *laevigata*, *Viola lutea*, *Festuca rubra*, and *Agrostis stolonifera* can be found on copper-bearing soils [21]. Another interesting example is the island of New Caledonia, where Ni hyperaccumulators are found in every taxonomic group, since most of the islands’ surface is covered by magma rocks rich in Mn, Ni, and Fe [20]. Europe is home to hyperaccumulator species such as *Thlaspi caerulescens* [22] and *Arabidopsis* *halleri* [23], which accumulate Zn and Cd, and *Agrostis capillaris* L., *Holcus lanatus*, *Calamagrostis epigejos* L., which accumulates As [24].

### 2.2. Parameters for Assessing Heavy Metal Resistance

For a plant to be classed as a hyperaccumulator, its heavy metal resistance must be assessed based on parameters such as bioaccumulation, tolerance, and contamination indices, as well as the translocation factor [25]. The bioaccumulation index indicates how efficiently plants accumulate metals and is expressed as the ratio of metal concentration in the plant relative to its surrounding soil content. The tolerance index indicates the extent to which the plant stops growing under culture conditions in contaminated soil. The contamination index is expressed as the ratio of the amount of plant dry matter in the contaminated soil relative to the amount of plant dry matter in the control medium. The translocation factor determines whether heavy metals are efficiently moved by the plant and is expressed as the ratio of metal content in the above-ground sections relative to the root metal content [26].

Using these indicators, a suitable plant can be selected, for example, in phytoremediation processes. Hyperaccumulators enable the rehabilitation of heavy-metal-contaminated soils which threaten human health [27]. Studying hyperaccumulators and remediation processes is therefore crucial.

## 3. Characteristics and Methods of Phytoremediation

The increasing number of heavy metals in the soil has contributed to the development of methods for soil purification, such as remediation [28]. Chemical, physical, and biological soil cleaning methods exist, and the latter is gaining increasing recognition for its satisfactory results and wide application range. Biological methods can be classified into bioremediation methods, which utilize the biological activity of microorganisms, and phytoremediation methods, which utilize higher plants with a high accumulation of heavy metals in tissues, also called hyperaccumulators. Examples of hyperaccumulators used for cadmium-contaminated soil phytoremediation are *A.* *halleri* and *S. nigrum* (Table 2) [29]. These plants must also have several other characteristics, including high biomass increase, tolerance for high soil heavy metal concentrations, low nutrient and water demand, rapid growth rate, and the ability to quickly move heavy metals to above-ground plant sections [30,31]. Important phytoremediation advantages include noninvasiveness, low cost, and environmental neutrality. The disadvantages include limited application range within a given area and a slow generation time for results [32].

Phytoremediation, for purifying contaminated soils, waters, and sediments, uses naturally occurring and genetically modified plants. Several different processes are associated with phytoremediation, such as: phytoextraction, phytoexcretion (phytovolatilization), phytodegradation, phytostabilization, and rhizodegradation [50].

Phytoextraction is a soil, water, and sediment purification method which utilizes the ability of plants to absorb toxic compounds through their roots and accumulate them in above-ground sections [51,52]. The ability to absorb and accumulate metals depends on a successful phytoextraction process, and choosing the correct plant is therefore important. This is a relatively low-cost and simple method, but the treatment process is long-lasting and limited to the soil surface layer. Phytoextraction can be categorized as continuous or assisted. Continuous phytoextraction involves plant heavy metal accumulation over the entire growing period, whereas for assisted phytoextraction, natural or synthetic substances supporting plant metal ion absorption (mainly copper and lead) are added to the heavy metal contaminated soil [51,52,53]. Natural hyperaccumulators are used for phytoextraction processes, and most of them belong to the Brassicaceae, e.g., *T. caerulescens*, *A. halleri*, and others such as *S. nigrum* [54].

Phytovolatilization involves plant heavy metal uptake, followed by transpiration and the eventual release of the absorbed substances into the atmosphere in a nontoxic form [55]. This technique is used to purify soils and aquatic environments from selenium, mercury, and arsenic; however, only mercury occurs in gaseous form. Transgenic plants are used for purifying mercury-contaminated soils, and contain the bacterial enzyme mercury reductase, which reduces Hg^2+^ to metallic mercury Hg^0^ [56]. This method is still largely unknown and creates controversy regarding the atmospheric release of toxic compounds [51,52,53,54,55,56].

Phytodegradation is a soil purification method where plants and microorganisms metabolize toxic compounds, reducing them to less-toxic substances [57]. These forms are incorporated into, and accumulate in, tissues. Phytodegradation also takes place in the plant root zone through enzyme secretion that degrades toxic substances [58].

Plants capable of immobilizing toxic compounds are used for phytostabilization. This means that toxic compounds do not spread to deeper soil layers, groundwater, or the atmosphere [59]. These plants secrete root exudates (organic acids and phenolic compounds) which bind to metal ions, making them less assimilable. The plants retain metals by root absorption and accumulation, or root surface adsorption. Plant characteristics required for phytostabilization include a high level of root metal accumulation in relation to shoots, rapid growth, a well-developed metal resistance, and an ability to excrete root exudates [60,61]. These characteristics can be found in *Typha latifolia* and *Sesbania rostrata* [62,63,64]. The plant’s ability to accumulate metals in its roots or rhizosphere, together with the fact that metals do not move into the shoots, protects herbivores by preventing toxic substances from entering their digestive systems, and also prevents the soil re-entry of metals when leaves drop to the ground [65].

Rhizodegradation utilizes plants that release root exudates into the rhizosphere, thus leading to more effective bioremediation via microorganisms [64]. Rhizosphere microflora benefit from the presence of plants that stimulate their growth and increase their metabolic activity, thereby increasing the effectiveness of toxic compound degradation [66]. The best-known rhizosphere microorganisms are bacteria and fungi that stimulate plant growth, rhizobia, and mycorrhizal fungi [67]. These organisms establish symbioses with plants through vitamin or hormone secretion, which stimulates plant growth, or through pathogen protection as plants secrete soil substances called rhizodeposits (e.g., organic acids, sugars, and enzymes). These rhizodeposits are natural pollutant analogues, which naturally selects for the microorganism population capable of breaking down toxic substances. Additionally, root exudates provide nutrients for the microorganisms [68,69].

Rhizofiltration is a method that removes pollutants from groundwater and wastewater and involves plant root sorption of heavy metal ions that have previously been precipitated, together with their subsequent root cell accumulation [70]. Of all the heavy metals, lead is most effectively absorbed from the soil by the root system. However, plants grown in hydroponic and aeroponic cultures are more efficient in rhizofiltration than naturally living aquatic plants [71,72].

## 4. Molecular Basis of Natural Heavy Metal Hyperaccumulation and Hypertolerance

### 4.1. Uptake and Transport of Metals by Endocytosis

**Figure 1:**
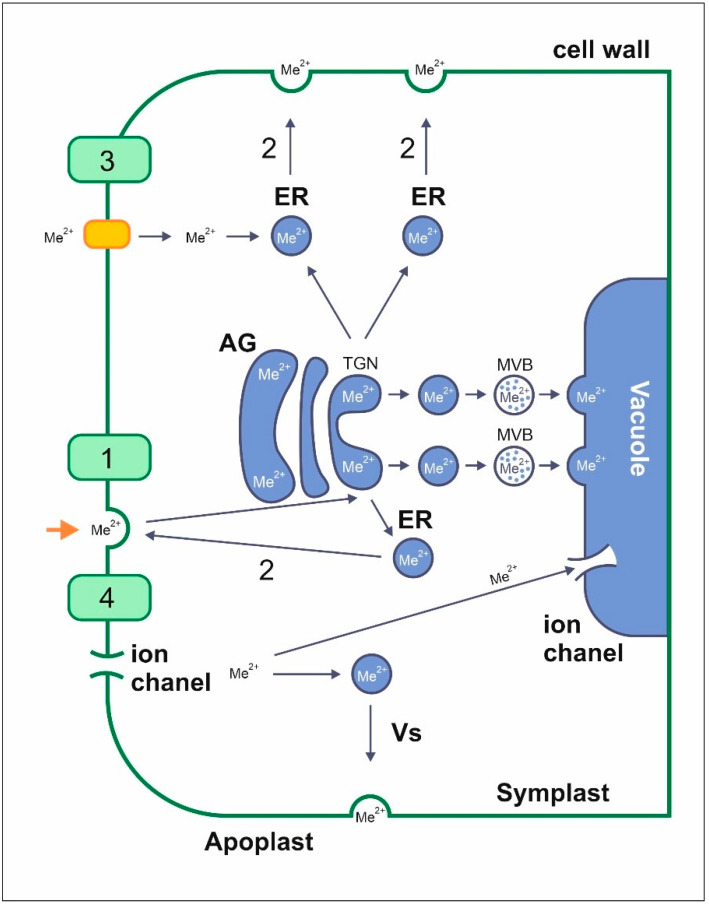
Cellular mechanisms of metal extraction/excretion and transportation through endocytosis—1, exocytosis—2, active transport—3, diffusion—4, and through ion channels; MBV—multivesicular body, Me^2+^—divalent metal, GA—Golgi apparatus, Vs—transport vesicles, TGN—early endosomes, RE—recycling endosomes [73].

Endocytosis it the reverse of exocytosis and comprises of: substance penetration from the cell wall and membrane into membrane depressions; early endosome formation and heavy metal transport; late endosome formation, known as multivesicular bodies, and substance transportation to a vacuole or the endoplasmic reticulum where, through exocytosis, the metals are transported to the cell membrane or wall (Figure 1) [73].

Endocytic aluminum uptake and transport in plants involves meristematic root cells, and Al accumulates in the vacuoles within the transition zone, where endocytosis occurs intensively [73,74]. However, in the proximal transition zone cells, no endocytosis occurs, and aluminum does not accumulate in the vacuoles, but instead accumulates within the cell walls [73,74,75].

Another example is Pb uptake via the endocytic pathway, whereby it is bound to a pectin fraction in the cell wall and then transported to the protoplast [11,75]. A low-esterified pectin fraction is a known cell wall component, and as a result of high Pb concentrations, the uptake of these fractions by endocytosis is much more intensive than for plants not treated with Pb. Most of the Pb is eventually deposited in the cell wall and its thickenings, as well as the vacuole. Thus, it is thought that the Pb expelled from the protoplast is not transported to it again, or that transport is impaired [11].

### 4.2. Transport of Metals to Different Plant Organs

Roots are the main organ that accumulates a high metal concentration to avoid damage to reproductive organs (flowers) or photosynthetic disorders (in leaves). To avoid zinc and lead transport to the shoots, Casparian strips in the endodermis block their passage, together with water, into the secretory and vascular tissues. Studies on plants highly contaminated with Pb showed it to be present only in the vegetative plant parts. The ovule and embryo sac, and the seeds developing from them, were Pb-free [11,74,76].

However, metal transport to above-ground sections can occur in the xylem. The Ni hyperaccumulator *Alyssum lesbiacum* showed a more efficient histidine synthesis, and together with Pb, formed a complex which was transported in the xylem vessels. Such a complex may be significant in terms of increased plant tolerance [2,76].

### 4.3. Uptake and Transport of Metals with Membrane Transporters

**Figure 2:**
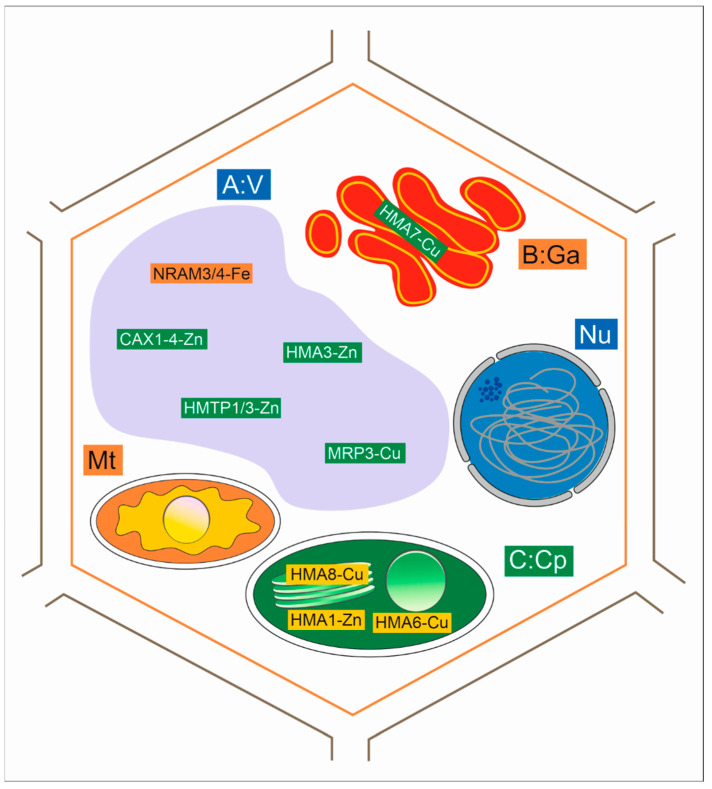
Heavy metals transporters. (A) Transporting metals to vacuoles with the use of NRAM3/4, HMA3, CAX1, MRP3, MTP1; (B) Transporting metals to the Golgi apparatus with the use of HMA7; (C) Transporting metals to tonoplasts with the use of HMA1 and HMA6 transporters, and to the thylakoid membrane with the use of HMA8 transporters [77].

Plants can maintain metal homeostasis due to transporters contained within the biological membrane (Figure 2). They are responsible for active metal ion transfer into the protoplast. The transporters can take up metal ions, as well as metal complexes with different substances, or transport them outside the cytoplasm. They are also found on organelle membranes [65,75,77].

Transporters are not specific, i.e., in addition to transporting essential/ballast elements in the plant, they transport harmful substances, e.g., too much cadmium, lead, or nickel [77]. Currently known plant membrane transporters are classified into various families, namely: P-type ATPase, ZIP (ZRT/IRT-like protein), YSL (yellow stripe-like), NRAMP (natural-resistance-associated macrophage protein), CDF (Cation Diffusion Facilitator), CAX (cation/H^+^ exchanger), COPT (specific high-affinity transporter), ABC (ATP-binding cassette), and IREG (iron-regulated transporter) [77].

Heavy metal ATPases transport substances across membranes using ATP hydrolysis energy [77]. In plants, heavy metal ATPases (HMAs) are classified into two groups. One group transports monovalent ions, such as Ag^+^ and Cu^+^, while the other group transports divalent ions, such as Zn^2+^, Cd^2+^, Pb^2+^, and Co^2+^. P1b-ATPases belonging to the ATPase subfamily that transport Cu^2+^, Zn^2+^, Pb^2+^, Co^2+^, Mo^2+^, and Cd^2+^. They consist of eight transmembrane segments, and an intramembrane motif, that binds metal ions to amine or carboxylic groups and distinguishes P_1b_-ATPases from other P-type ATPases [77,78]. In *A. thaliana*, eight metal-transporting ATPases were discovered, namely AtHMA1-8 (Heavy Metal ATPase). Four are Cu^+^-ATPases (HMA5-8) and three are Zn^2+^-ATPases (HMA2-4), as well as HMA1, which also transports Cu^+^; however, the latter’s’ binding site is different from HMA5-8 [77,78,79].

HMA7 is responsible for transporting Cu^+^ to the Golgi apparatus, while HMA6, 8, and 1 transport Cu^+^ to the chloroplasts. HMA5 is involved in root copper detoxification. Zn^2+^-ATPases are found in the plasma membrane, and their increased expression has been observed in root and shoot vessels. Excessive HMA4 expression causes increased Zn^2+^ transport from the roots to the shoots. Considering their distribution, these are involved in metal unloading to/from the phloem, or metal transport to/from the xylem. AtHMA4 also participates in cell detoxification, and in cases of elevated Cd^2+^ concentration, this transporter removes it from root cells and transports it to the shoots [75,77,78,79]. In vascular tissue, metals are transported by forming organic acid–metal complexes or metal–nicotianamine [79].

In *A. halleri*, an AhHHMA4 transporter is located in the cell membrane, which participates in cytoplasmic cadmium and zinc removal to outside the protoplast, while AtHMA3 participates in Zn^2+^, Co^2+^, and Pb^2+^ ion sequestration by transporting them to the vacuoles [65,79].

The heavy metal carrier ATPase 3 (HMA3) is located in tonoplasts and is responsible for the vacuolar sequestration of metals. In rice, HMA3 limits Cd deposition in seeds and leaves. In *A. thaliana*, HMA3 increases Cd, Zn, and Co accumulation and tolerance [11]. OsHMA3 of *O. sativa* is responsible for Cd transport in root vacuoles. The loss of HMA3 function in *A. thaliana* and *O. sativa* reduces cadmium levels in root vacuoles, thereby causing high Cd accumulation in the above-ground sections [77,78].

Studies on HMA4 have shown that excessive Zn accumulation, and full Cd and Zn hypertolerance in *A. halleri*, depend on the HMA4 metal pump, and the increased expression of this transporter in *A. halleri* is due to a combination of modified cis-regulatory sequences and copy number expansion compared to *A. thaliana* [79].

ZIP transporters are membrane proteins that transport divalent metals and are responsible for maintaining homeostasis by transporting these metals to the protoplast. The ZIP family includes transporters responsible for Zn^2+^ (ZRT, zinc-regulated transporters) and Fe^2+^ (IRT, iron regulated transporters) transport [65,75]. Their highest expression can be seen in the root epidermis. ZIP proteins play an important role in metal accumulation in the Zn and Cd hyperaccumulators *T. caerulescens* and *A. halleri*. The IRT1 transporter, located in *A. thaliana*, is responsible for metal ion uptake, such as Fe^2+^, Zn^2+^, Mn^2+^, Co^2+^, and Cd^2+^ [77].

Among the Zn^2+^ transporters in *A. thaliana* are ZIP4, ZIP9, and ZIP10, while IRT3 is responsible for transporting zinc to the cytoplasm, and ZIP1, ZIP3, and ZIP12 are responsible for intracellular transport through the membranes [75,77].

The expression of ZIP genes (*ZTN1* and *ZTN2*) in *T. caerulescens*, and *ZIP6* and *ZIP9* genes in *A. halleri* in nonaccumulating plants, is regulated by Zn level and occurs only in cases of its deficiency, while in hyperaccumulators, the expression of these genes is independent of the Zn level and constantly maintains a high value [65,77]. The transporters CAX are among the best characterized secondary metal transporters in plants. The CAX protein group is one of five subfamilies and belongs to the CaCA (calcium/cation antiporters) family, a group of membrane proteins that transport cations using proton gradients. The CAX protein forms a transmembrane domain of an α-helix structure characteristic of the CaCA family and subfamily, namely the CaD domain (calcium domain). This is located in the CAX1 protein and is essential for calcium transport. Studies on transgenic tobacco plants synthesizing AtCAX3 (calcium/cation antiporters) proteins with the AtCAX1 CAD domain showed a high Ca accumulation, thus gaining resistance to this element. CAX transporters differ in their transporting ability due to lacking the amino acid sequence conservativeness of CaD. As for the regulatory/autoinhibitory domain, it occurs in the N-terminal regions, and is responsible for regulating CAX function. The acidic amino acid motif serves an important function in calcium ion bonding. The manganese domain is a region responsible for manganese ion transport, which is characteristic of CAX2 and is made of cysteine–alanine–phenylalanine. This region was also identified in the AtCAX5 and AtCAX6 transporters of *A. thaliana*, and in ZCAX2 in *Z. mays.* For the c-1 (occurring on the vacuolar side) and the c-2 (occurring on the cytoplasmic side), loop mutations in their genes inhibit or reduce Ca^2+^ and Mn^2+^ transport. They function as filters, i.e., they are responsible for cation uptake or selection. The D-domain is characteristic for the CAX subfamily and is responsible for regulating the transport of calcium and other cations by CAX proteins due to cytoplasmic pH changes [80].

CAX transporters maintain calcium homeostasis. In studies on transgenic tobacco, CAX1 protein overexpression caused calcium deficiency symptoms. However, studies on *A. thaliana* showed that the expression of genes encoding CAX2 and CAX transporters is regulated by high Cd, Mn, and Ni concentrations [80].

The NRAMP family of transporters belong to the integral membrane proteins that transport divalent metal ions, mainly Fe^2+^. They are responsible for moving metals into the cytoplasm from the cell wall and from the vacuole. The AtNRAMP1 (natural resistance macrophage protein (1)) transporter, located in *A. thaliana*, transports Fe^2+^ ions from the soil. Meanwhile, AtNRAMP3 (natural resistance macrophage protein (3)) and AtNRAMP4 (natural resistance macrophage protein (4)) transfer Fe ions to the vacuole, where the metal deposited in the vacuole can be recovered during germination. Proteins from this family can also transport Cd^2+^, Zn^2+^, Mn^2+^, and Ni^2+^. For example, the TjNRAMP4 transporter in *T. japonicum* has the sole responsibility of transporting Ni to the cytoplasm [77,81].

The NRAMP protein is made of 12 transmembrane helices with a characteristic, long hydrophilic C-terminal of a polypeptide chain, which faces the cell interior [81].

The YSL proteins belong to the oligopeptide transporting protein family. They are involved in metal uptake and transport, in the form of complexes, in monocotyledonous and dicotyledonous plants. A single oligopeptide chain consists of about 700 amino acids, of which the N-terminal is rich in glutamate and aspartate. In monocotyledons, YSL proteins are located in the roots, where they transport metals, mainly iron in a complex with phytosiderophores. Low iron levels in maize activate phytosiderophore synthesis. In dicotyledons, metals are transported from the apoplast to the cytoplasm in a complex with nicotianamine. Different YSL transporter expressions have been observed in rice and is dependent on the Fe level. In the case of an Fe deficiency, excessive OsYSL2 expression occurs in rice seeds and leaves, and OsYSL15 in roots, while OsYSL13 gene expression is not dependent on this. OsYSL proteins transport iron and manganese complexes with nicotianamine, and their high expression in cells accompany the vasculature, through which they participate in metal transport regulation. In *A. thaliana*, eight genes have been identified to encode YSL proteins that transport Fe, Zn, and Cu with nicotianamine. The AtYSL2 transporter is located in the leaf cells surrounding the vessels and is responsible for transporting iron complexes from the roots to the vascular bundles. The proteins AtYSL1 and AtYSL3 are responsible for delivering Fe^2+^ ions to the seeds. AtYSL2 gene expression occurs in cases of optimal iron levels and the stimulation of its expression in the presence of Cu^2+^ ions. In *T. caerulescens*, the TcYSL3, TcYSL5, and TcYSL7 genes are responsible for the transport of a complex of metal ions with nicotianamine from the cell wall to the cytoplasm and have been identified in vascular system cells [77,81].

YSL transporters can be separated into two classes. The first is characteristic for Poaceae plants and contains the ZmYS1 transporter in *Z. mays*, OsYSL15 in *O. sativa*, and HvYS1 in *H. vulgare*. They are responsible for transporting Fe ion phytosiderophore complexes from the soil to the root cells. The second class is responsible for intracellular transport and distribution of metal complexes with nicotianamine or phytosiderophores and includes AtYSL1-4 and 6 in *A. thaliana*, OsYSL2, 6, 16, and 18 in rice, and HvYSL2 and 5 in barley. Research on a new gene isolated from *S. nigrum*, SnYSL3, has shown that its expression occurred in cases of excess cadmium and increased over time, but was not induced by the presence of Fe. This may indicate that the SnYSL3 gene does not absorb Fe from the soil. Excessive expression occurred in vascular bundles, but this gene’s transport activity is also significant in other plant parts. The SnYSL3 protein transports a metal–nicotianamine complex and plays an important role in the plant’s stress response caused by excess cadmium [82].

CDF family proteins are responsible for Zn^2+^, Co^2+^, Cd^2+^, Mn^2+^, and Ni^2+^ transport. CDF proteins transport metal ions through membranes based on a H^+^/Me antiport, from the cytosol to the organelles, or outside the cell [65,75]. CDF transporters are located in the cell membrane, as well as intracellular membranes. The first identified transporter was ZAT1 (zinc Arabidopsis transporter 1), later named AtMTP1 (metal tolerance protein 1), in *A. thaliana*, which is responsible for transporting zinc to the vacuole, i.e., sequestration. MTP1 transporters and AtMTP1 homologues have also been found in the cell membrane of the hyperaccumulator *T. goesingense*. AgMTP1t1 is responsible for Zn^2+^, Co^2+^, and Cd^2+^ transport, and AgMTP2t2 is responsible for Ni^2+^ transport, which contributes to increased plant tolerance towards these elements. In the hyperaccumulator *A. halleri*, AhMTP1-3 have been described in the tonoplast, which transport zinc ions to the vacuole. In the *Populus trichocarpa* x *P. deltoides* hybrid, MTP1 transporters have been identified which are located in vacuole membranes and are responsible for Zn^2+^ detoxification [64,74,76]. CDF proteins are made up of four hydrophilic helix polypeptides (1, 2, 5, 6) which form the protein core and two hydrophobic helices that are oriented outwards. Helices 2, 5, and 6 are rich in aspartate residues and a signature domain is also characteristic for this family. The cytoplasm domain between helices 4 and 5 is rich in residual histidine and may be zinc-binding [77,83].

COPT are responsible for Cu^2+^ ion collection and transport. Five COPT transporters (1–5) have been found in *A. thaliana* [65,83], with a higher shoot activity. COPT proteins occur in plasma membranes and transport copper from extracellular spaces to the cytoplasm or vacuole. The COPT protein level is regulated by copper ions [75,83].

IREG transporters are nonselective cell membrane channels, which are permeable to mono- and divalent metal ions. They are homologous to cyclic nucleotide channels in animals. NtCBP4 is one of the IREG family transporters and is located in *N. tabacum*. In transgenic plants, IREG transporter activity is high and contributes to an increased Ni^2+^ tolerance and uptake reduction, as well a sensitivity to, and accumulation of, Pb^2+^. The most well-known transporter is LTC1 (lipid transfer at contact site 1), which is capable of moving Cd^2+^, Ca^2+^, Pb^2+^, K^+^, and Na^+^; it is located in the cell membrane. It detoxifies the plant of cadmium and regulates various cellular processes, with detoxification dependent on soil Ca^2+^ concentration. When the calcium concentration is low, LTC1 may contribute to an increased root Pb^2+^ accumulation [75,77,84].

ABC transporters derive energy from ATP hydrolysis by transporting ions, lipids, carbohydrates, peptides, antibiotics, and xenobiotics, among others. They can be classified into (1) the MRP (multidrug resistance-associated protein) subfamily, which are proteins involved in toxin resistance, (2) the ATM (ABC transporter of the mitochondria) subfamily, which carry mitochondrial proteins, and (3) the plant PDR (pleiotropic drug resistance) subfamily associated with pleiotropic toxin resistance. The AtMRP3 transporter identified in the hyperaccumulator *A. thaliana* is responsible for the transport of cadmium ions and its complexes with other substances, and its activity occurs when Cd^2+^ is present in the plant, or, to a lesser extent, Cu^2+^ or Zn^2+^. AtATM3 (ABC transporter of the mitochondria (3)) is also responsible for transporting cadmium ions and its complexes with other substances, e.g., glutamine synthetase with Cd^2+^. AtPDR8 (pleiotropic drug resistance (8)) is located in the root cell membrane of *A. thaliana* and is responsible for the transport of Cd^2+^ and Pb^2+^ ions, and their complexes, from the protoplast, thus decreasing cytosol metal levels and increasing the plant’s cadmium and lead resistance. AtPDR12 (Pleiotropic Drug Resistance12) is found in the cell membrane of *A. thaliana* and acts as a pump that transports lead ions and its complexes from the cytoplasm, thus increasing the plant’s resistance [77,85].

ABC transporters consist of three structural types. The full transporter contains two transmembrane and two nucleotide-binding domains. Half of the number of ABC transporters are comprised of one TMD (transmembrane domain) and one NBD (nucleotide-binding domain). Transporters also exist that do not contain a TMD domain, and instead consist of two NBD domains. The NBD domain is present in all structural types and contains characteristic motifs, including the D loop, which is responsible for keeping dimers together, the H switch loop, which interacts with the transmembrane domain, the Walker A and Walker B motifs, which form the P loop and is associated with ATP, and finally the characteristic Q and H loop, as well as the signature motif, which only occurs in ABC proteins, thereby making it possible to distinguish them from other ATPases. ABC transporters can be classified according to the TMD domain presence, function, protein solubility, and amino acid sequence. One classification is the division of transporters into eight subfamilies (A–G, I), of which ABCH subfamilies have not been identified in plants. The ABCE and ABCF subfamilies belong to soluble proteins devoid of the TMD domain. One study has shown these transporters to be important in sequestration, and they impart herbicide resistance, since they can sequester glyphosate in vacuoles [77,85].

### 4.4. Strategy for Avoiding Heavy Metal Uptake

#### 4.4.1. Symbioses with Rhizospheric Microorganisms

In order for a plant to properly develop under stress conditions, such as the presence of heavy metals, numerous rhizospheric microorganisms are needed to stimulate plant development. Known rhizospheric microorganisms are the symbiotic papillary bacteria of legumes, bacteria, and fungi, which stimulate plant growth and mycorrhizal fungi. These microorganisms can release substances into the rhizosphere that change the soil physical and chemical nature, thus modifying soil metal bioavailability [86].

Fungi form a symbiotic relationship with the plant root system (mycorrhizae). Mycelia that develop on the root surface are known as external mycorrhizae (ectomycorrhizae), while mycelia that develop inside the root are known as arbuscular mycorrhizae (endomycorrhizae). A characteristic structure found in ectomycorrhizae are the muffs produced by the mycelium. The mycelium penetrates the root cortex cells and create a Hartig net, i.e., a channel for substance exchange between the fungus and plant. Other characteristic elements are rhizomorphs and loose hyphae, which, together with muffs, store and exchange mineral salts and water. Trees characterized by increased heavy metal concentrations, e.g., *P. sylvestris*, form ectomycorrhizae with *Scleroderma citrinum*, *Lactarius rufus*, *Rhizopogon roseolus*, and *Amanita muscaria*. They accumulate heavy metals in root or rhizomorph muffs, which contribute to the nontransportation of metals to above-ground plant sections. Ectomycorrhizal fungi secrete exudates to facilitate metal ion bonding in muffs, and to limit metal ion penetration into the root cells, by attaching them to polysaccharides in the fungal hyphae cell walls. Approximately 120 fungal species from the order Glomales participate in arbuscular mycorrhizae, primarily increasing the soil nutrient bioavailability. Endomycorrhizae occur in herbaceous plants as well as in ash, maple, and fruit trees [87].

Mycorrhizal fungi significantly increase heavy metal plant resistance, but these defense mechanisms may vary depending on the fungus, the metal type, and its concentration. The pine tree ectomycorrhizal fungus, *Thelephora terrestris*, increases plant zinc levels, while *Glomus caledonium* increases the Cu^2+^ concentration in maize, but only when these metals have a low soil level. The fungi limit their uptake when these metals reach high concentrations. Arbuscular mycorrhizal fungi also play a protective role, an example of which is *Glomus intraradices*, and reduce toxic cadmium effects in *P. sativum*. Endomycorrhizae also reduce stress caused by water scarcity: the fungi produce more metal-binding substances and improve nutrition with mineral salts. Plants in symbiosis with fungi also contain elevated root phenol levels, which, in the presence of high cadmium concentrations, causes cell wall stiffening, which makes the penetration of aqueous Cd^2+^ solutions more difficult [87,88].

Bacteria stimulating plant growth and development, i.e., PGPR (plant growth-promoting rhizobacteria), increase plant heavy metal tolerance. They affect metal mobility and availability through the release of chelating agents, acidification, phosphate solubilization, and changes in the rate of metal oxidation. Examples of PGPR bacteria that improve plant growth in areas with high zinc and lead levels are *Actinobacteria*, *Streptomyces*, and *Pseudomonas* [26].

#### 4.4.2. Release of Substances into the Soil and Immobilization of Metals

Metal chelators are substances secreted by plants into the soil that form insoluble metal complexes, i.e., they immobilize them while simultaneously protecting the plant against toxic substance uptake. Such substances are organic acid anions (e.g., citrate), which, when activated by the presence of Al^3+^ in the soil, are released through the cell membrane anion channel and secreted into the rhizosphere. Where the aluminum ions are most concentrated, the metal is bound and immobilized. In addition to organic acids, plants can also secrete phenolic compounds (e.g., catechin) into the rhizosphere, thereby improving the Al^3+^ immobilization mechanism, but only if the plant has first been treated with silicon [89]. When there is an excess of metals, plant roots also secrete compounds such as callose, histidine, or mucus, which, as with organic acids, bind metals and immobilize them, thus protecting them from the stress caused by excessive metal concentrations [31].

Other mechanisms that limit heavy metal uptake, i.e., stress factor avoidance mechanisms, are increased pH, which contributes to decreased metal uptake, and the formation of a root oxidizing zone, which involves metal oxidizing, thereby reducing their availability [31].

#### 4.4.3. Changes in Cell Wall Permeability and Its Modifications

The cell wall is the first barrier separating the plant from the external environment and is therefore important in protecting the plant against heavy metal penetration. Cell wall metal accumulation contributes to increased plant tolerance to metals and partakes in detoxification. In studies on different soybean varieties with high cadmium concentration tolerance and metal sensitivity, differences in cell wall composition were found. It was shown that 48.5–75.5% of Cd had accumulated in the cell wall. Pectin, hemicellulose, and cellulose are the main cell wall components and are mostly polysaccharides, which, apart from monosaccharides, may contain uronic acids. Pectin and hemicellulose, as main components, contain uronic acids, which bind to heavy metals [90].

The binding of metals in a cell wall may depend not only on differences in its composition, but also on the chemical form of pectin, which in turn depends on the degree of esterification. A high degree of demethylated esterification contributes to increased Al^3+^, Cd^2+^, and Cu^2+^ binding, and can be determined by pectin methylesterase activity, which transforms pectin into a loose carboxylic structure with the release of methanol and protons [90,91].

Moreover, under the influence of high Cd levels, lignin in the secondary cell wall causes wall hardening, i.e., its lignification, which protects the plant from being crushed and facilitates water and mineral salt conduction. Lignin is an important cell wall component because it forms complexes with metals such as Cd, Cu, Zn, as well as cellulose, and the number of these complexes affects cell wall permeability, thus affecting heavy metal tolerance and uptake [89]. Sensitive plants use lignin stored in cells as a barrier against cadmium penetration into the plant. When a plant is treated with high Cd concentrations, root genes increase and are overexpressed; genes involved in lignin biosynthesis and genes associated with cell expansion, in *A. thaliana*, a plant that is sensitive to Cd stress, while in *N. caerulescens*, the expression of these genes remains at a high and constant level [92].

Another mechanism that is also an indicator of plant stress is the production of components that create a barrier against metal penetration into the plant, such as callose, cutin, and lignin. If Al, Co, Ni, Zn, and Pb concentrations are high, the plant synthesizes callose on the outer cell membrane surface within a few minutes, thereby producing a layer which is impermeable to metals. Moreover, all these modifications cause the primary cell wall to assume the character of a secondary wall due to changes such as an increased number of transverse bonds between polysaccharide molecules, lignin production, phenolic substances, and a greater cell wall thickness. These changes contribute to the formation of a structure which is flexible and permeable to aqueous solutions, and is compact, rigid, and thick, and consequently impermeable to metals [65].

### 4.5. Strategy of Stress Tolerance

Heavy metal tolerance mechanisms are activated when metals penetrate the cells and where their presence is most dangerous for the plant. One of these mechanisms consists of reducing the activity of cell membrane transporters that uptake metal ions from the rhizosphere, which is in turn caused by the increased expression of genes encoding the transporters, located in the tonoplasm, and causing metal storage in the vacuole [93].

#### 4.5.1. Chaperones

Metals such as Hg, Pb, or Cd can interact with proteins and DNA, and can damage nucleic acids and inhibit protein folding. The inhibition of protein folding under stress conditions leads to protein inactivity, thereby making them nonfunctional. Therefore, plant cells activate proteins, i.e., chaperones, which can repair these proteins and protect them from developing when heavy metal are in excess [94].

Metals that are toxic or in excess within the cell can be bound by chaperone proteins. These proteins then transport heavy metal ions to where they can be built into specific molecules or structures and perform specific functions [64]. The tolerance mechanism, based on the action of these proteins, only works for metal ions that participate in cellular metabolism. The mechanism of Cu^2+^-binding and transportation in *A. thaliana* is similar to that found in yeast. In *A. thaliana*, copper is absorbed into the cell cytoplasm by COPT membrane transporters, where it is bound to the chaperone CCH (copper chaperone). The copper complex from CCH is transported to Golgi apparatus vesicles. Hereafter, Cu^2+^ passes to the follicular lumen via transporters, and is incorporated into the ERT1 (ethanol regulator of translation 1) protein, which is transported and built into the cell membrane where it acts as an ethylene receptor. The mechanism by which copper ions are delivered to the mitochondria, with the help of the AtCOX17 (cytochrome C oxidase copper chaperone) chaperone, is also known. An increased activity of these proteins has been observed when copper ions are excessive, as well as bacterial infections and NO or H_2_O_2_, which disturb mitochondrial functioning [95].

#### 4.5.2. Chelation of Heavy Metal Ions

To prevent toxic heavy metal effects in the cytoses, plant cells have developed a number of detoxification mechanisms, of which metal ion chelation is the main mechanism. Chelation involves the binding of metal ions by cysteine-rich peptides to form a nontoxic complex, which is sequestered in a vacuole to prevent the free movement of metals in the cytosol. The most important classes of cysteine-rich peptides that are involved in chelation are the synthesized enzymes phytochelatins (PC), known as class III metallothioneins (MT), as well as class I and II metallothioneins, which are primary gene products. The heavy metals found in the cytosol can also be bound by glutathione, organic acids, nicotianamine, and amino acids [96].

Metallothionein (MT) is a cysteine-rich protein with a low molecular weight and is able to bind metal ions. Six MTs have been identified in *A. thaliana*, belonging to four forms classified by the type of cysteine residue. These are MT1a, MT2a, MT2b, MT3, MT4a, and MT4b. Most of these MTs contribute to Cu tolerance; however, MT1, 2, and 3 are also able to bind Cd, and MT4 can bind and accumulate Zn. In cases of high Cd concentrations in *A. thaliana*, MT1a binds and accumulates cadmium, thereby increasing the plant’s tolerance to the metal. *N. caerulescens* has shown a higher level of MT1 and MT2 compared to *A. thaliana*, which makes it more tolerant to Cd and able to store higher Cd concentrations [92,94].

Phytochelatins are low-molecular-weight peptides consisting of glutamic acid, cysteine, and glycine. These peptides are synthesized from glutathione (GSH) by phytochelatin synthase (PCS), an enzyme which requires post-translational modification to be active [97,98]. Phytochelatins have a high affinity for binding heavy metals when their concentration is toxic. Phytochelatins are produced in response to stress caused by high metal concentrations and can therefore act as biomarkers for early stress identification. PCs are produced in the cytosol, where they are first bonded to metal, whereafter the metal phytochelatin complex is then transferred to the vacuole. It is possible that ABC family transporters or magnesium-dependent ATP carriers are involved in this transport. PC synthesis takes place due to different MT1, 2, 3, and 4 levels, as well as in the presence of Cd^2+^ ions, which stimulate PC synthesis by up to six times as much as Cu^2+^ or Zn^2+^ ions. Additionally, phytochelatins are the first to be synthesized and accumulated within the roots. Moreover, phytochelatins increasingly accumulated in *S. nigrum* L. treated with high Cu concentrations, which contributed to binding and immobilization of excess copper, making its transport to the shoots impossible [96]. In the case of nonhyperaccumulator plants and those intolerant to Cd, phytochelatins play an important role in maintaining the homeostasis of this metal, whereas in plants capable of hyperaccumulation and hypertolerance, this mechanism is less significant; in this case the Cd sequestration mechanism is more effective [92].

Glutathione is a tripeptide containing a thiol group, which makes it capable of capturing metals, and it is also a cellular antioxidant and signal molecule for reactive oxygen species (ROS). An increased expression of genes responsible for glutathione synthesis has been observed in *A. thaliana* treated with cadmium, which increases its tolerance to this metal, while a reduction in GSH levels contributed to a decreased Cd tolerance. Rice studies have found that plants which can tolerate Cd have higher glutathione levels than plants sensitive to this metal. When the cytoplasmic cadmium concentration is high, a GSH–metal complex forms, which has a higher affinity for phytochelatin synthase than free heavy metal ions, and which causes its activation. Activation is achieved by protein folding and conformational changes, resulting in phytochelatin synthesis. GSH plays an important role as a substrate in phytochelatin synthesis [94,97].

Nicotianamine (NA) is a nonprotein amino acid which binds metals such as Zn, Ni, Co, Mn, and Fe. NA is synthesized by the enzyme nicotianamine synthase (NAS), which has a different number of genes in different species. The expression of these genes occurs when heavy metals are in excess, and are responsible for increased NA concentrations; thus, they help to maintain metal homeostasis. Only one NAS gene is known from Solanaceae plants, while the Poaceae has three genes, and the Brassicaceae has four, namely: NAS1, 2, 3, and 4 [92].

Metal–NA complexes can be transported through the cell membrane due to membrane transporters from the YSL family. In tonoplasts, the ZIF1 (zinc-induced facilitator (1)) transporter is responsible for transporting NA from the cytoplasm to the vacuole, where the metal–NA complex is formed. The ZIF1 gene is expressed in *A. thaliana* when zinc is in excess, which results in increased Zn transport to root cell vacuoles, where a NA–Zn complex is formed, thereby making its transport to the shoots impossible, where zinc is more toxic [92].

#### 4.5.3. Production of Heat-Shock Proteins

Heat-shock proteins (HSP) are characterized by a conservative primary structure, and are produced in response to abiotic stress, such as high temperature, oxidative stress, UV radiation, and high heavy metal concentrations. HSP proteins can be categorized into five classes: the HSP60 family (chaperonins), the HSP70/DnaK family, the HSP90 family, the HSP100/ClpB family, and the small heat shock proteins (sHSP) family [98,99,100,101,102,103]. HSPs are found in all prokaryotic and eukaryotic organisms, whereas plants have the highest number of these proteins of all eukaryotes.

In rice, 25 HSP proteins have been identified, while 38 have been identified in soybean and 21 in *Arabidopsis* [104]. Under the influence of stress caused by heavy metals or other harmful conditions, proteins may be damaged/denatured; at this point, HSP proteins are activated, which prevent the aggregation of denatured proteins and contribute to their refolding. In this way, HSP proteins act as chaperones. Additionally, as chaperones, they participate in the modification of newly synthesized proteins, change the spatial conformation of abnormally folded proteins, and play a role in the proteolytic degradation of proteins [100,101]. Moreover, stress caused by excessive Cd amounts induces the expression of HSP70 and HSP60 family proteins [92].

The best known and most conservative is the HSP70 protein family. It is constructed from three domains, namely: the N-terminal domain, interdomain, and C-terminal domain. The C-terminal domain binds to the substrate, while the N-terminal domain binds ATP. Genes encoding HSP70 proteins have been identified in many plant species, including *O. sativa*, *S. oleracea* L., and *A. thaliana*, with 18 identified in *A. thaliana* and 32 in *O. sativa*. A total of 61 HSP70 genes were identified during the analysis of HSP proteins in *N. tabacum* [103].

### 4.6. Expression of Genes Associated with Heavy Metal Tolerance

Genetic control is one of the mechanisms for the development of plant heavy metal tolerance. Heavy metal tolerance can be associated with the overexpression of associated genes or with the repression of these genes. There are three models of genetic control mechanisms associated with heavy metal tolerance. The first model consists of one main gene, which can interact with other genes capable of changing the expression of the main gene. The next model is a multigenous model, where there are a small number of genes, with each gene having an equal effect on tolerance. The polygenic model is a mechanism where metal tolerance is associated with a different number of genes, and each of them has a small influence on the tolerance variation [31].

Recent studies have shown a strong influence of microRNA (miRNA) in the modulation of gene expression. This is a nonprotein RNA constructed from about 21 nucleotides, which performs the role of gene expression modulation at the post-transcriptional level. The miRNAs are involved in the abiotic stress response. Under stress conditions, plants also activate the mechanism of miRNA-mediated post-transcriptional regulation. Rice studies showed changes in the expression of 19 miRNAs caused by excessive Cd levels. However, a high expression level was only observed for miR528, and the level of the remaining miRNAs was reduced [101,102].

Mature miRNAs attached to the RISC (RNA-induced silencing complex) cause the target mRNA expression to be downregulated by chopping up, degrading, or blocking translation. Studies on *Ricinus communis* L. in conditions of metal toxicity demonstrated miRNA level changes, which translate into expression level changes of the target genes. The levels of seven miRNAs involved in the stress response caused by heavy metals have been studied. It was shown that, under elevated Ni^2+^ conditions, the level of miR398 was reduced and the target gene overexpressed. It is also known that most plant miRNAs are regulated by transcription factors (TF) [101,102].

Transcription factors act as regulators in response to stress by regulating the expression of genes associated with stress factor tolerance. Under toxic cadmium conditions, TF regulates the expression of target genes such as CaPF1 and HsfA4a. Studies on TFs of the WRKY family have shown that the transcription of WRKY12 is inhibited in the presence of high Cd levels. The WRKY family is involved in the stress responses of many plants, e.g., in WRKY53 in *T. caerulescens* and WRKY4 in *Z. mays* [105].

Many genes are involved in heavy metal stress responses. In *B. nigra*, 88 genes with a different level of mRNA in the roots and 24 genes in the shoots were identified in response to high Cd levels. These genes were associated with metal accumulation, chelation, and transport [106]. In the presence of high heavy metal levels, the expression of genes encoding for membrane transporters increased, e.g., the ZAT1 gene involved in zinc transport. The increased expression of these genes simultaneously causes a decrease in the expression of genes associated with metal uptake from the environment. The presence of heavy metals also contributes to the increased expression of genes encoding for metal chaperones and chelators, such as phytochelatins or metallothioneins. All studies confirm that metal homeostasis in plants is closely related to the expression of the respective genes involved in heavy metal stress responses [65].

## 5. Conclusions

Thanks to the increasingly rapid development of molecular techniques, it is possible to perform more accurate heavy metal hyperaccumulator analyses. They allow the discovery of the unknown mechanisms that induce plants to become hyperaccumulators, thus creating more opportunities for more efficient phytoremediation of contaminated soils (see Appendix A).

## Figures and Tables

**Table 1:** Types of plant strategies allowing them to adapt to the presence of heavy metals.

#	Type of Strategy	Description
1	Strategy for avoiding heavy metal uptake	The formation of symbioses with rhizospheric microorganisms which stimulate plant growth under stress conditions [7]. Developing mechanisms which prevent heavy metals from entering the root cells by releasing substances into the soil that immobilize metals [8]. The formation of a rhizosphere oxidation zone which oxidizes metals, thus reducing their solubility and availability [9]. A rhizospheric pH change, whereby an alkaline environment reduces metal availability [10]. Reduction in cell wall permeability, which forms a barrier against protoplast metal penetration [11]. Cell wall modification by creating surface components (callose, lignin, cutin) or by increasing the wall’s metal accumulation capacity [12].
2	Strategy of plant tolerance to heavy metals (ion uptake and neutralization)	Change in expression of genes encoding tonoplast transporters, responsible for metal ion uptake and sequestration, contributes to an activity reduction [13]. Binding of metal ions (involved in metabolism) by proteins—chaperones and their transport to cellular compartments which use the ions, e.g., incorporating them into enzymatic molecules [14]. Chelation of heavy metals into the cytosol by metallothionine classes I and II, organic acids, and the amino acids (histidine), glutathione (GSH), phytochelatin (PC), and nicotianamine (NA), followed by transfer of complexes to the vacuole or cell wall [15]. The production of heat-shock proteins (HSP), with a regenerative function, that efficiently and quickly repair damage [12].

**Table 2:** Selected heavy metal plant hyperaccumulators used in phytoremediation.

Heavy Metals	Hyperaccumulators	Methods of Phytoremediation	References
Cd, Cu, Pb, Zn	*Salix viminalis*, *Salix fragilis*	phytoextraction	[33,34,35]
Cd	*Ricinus communis*, *Thlaspi caerulescens, Arabidopsis halleriandSolanum nigrum* L.	phytoextraction	[36,37]
Cd, Pb, Zn	*Zea mays*	phytoextraction “phytoattenuation”	[38]
Cd, Cu, Pb, Zn	*Populus deltoides*, *Populus nigra*, *Populus trichocarpa*	phytoextraction, phytostabilization	[35,39]
Cd, Cu, Ni, Pb, Hg	*Jatropha curcas* L.	phytoextraction	[40]
Cu, Pb, Fe	*Eichhornia crassipes*	rhizofiltration	[41]
Hg	*Populus deltoides*	phytovolatilization	[42]
Se	*Brassica juncea*, *Astragalus bisulcatus*, *Astragalus racemosus*, *Cardamine hupingshanesis*	phytovolatilization	[43]
Zn	*Populus nigra*, *Populus canescens*	phytoextraction	[44]
Ni	*Alyssum murale*, *Berkheya coddii*, *Thlaspi goesingense*	phytoextraction	[45]
Pb, Cd, Cu, Ni, Zn, Cr	*Thlaspi caerulescens*, *Brassica juncea*, *Pteris vittata*, *Arabis paniculata*, *Lolium italicum, Alyssum heldreichii*	rhizofiltration	[46]
Cu, As, Cd, Pb, Zn	*Piptatherum miliaceum,* *Euphorbia* sp., *Atriplex lentiformis*	phytostabilization	[47,48,49]

## Appendix A

**Table A1:** Selected genes of transporters isolated from plants involved in heavy metal uptake.

Genes	Plant	Elements	References
**ZIP gene family:**			[106,107,108,109,110,111]
*TcZNT1*	*Thlaspi caerulescens*	Zn	
*AhIRT3*	*Arabidopsis halleri*	Fe	
*AtZIP1-3* *AtZIP5-7* *AtZIP9-12* *AtIRT1* *AtIRT2* *AtIRT3*	*Arabidopsis thaliana*	Fe, Mn, Cu, Zn, Cd	
*MtZIP1* *MtZIP3* *MtZIP4* *MtZIP5* *MtZIP6* *MtZIP7*	*Medicago truncatula*	Mn, Fe	
**P-type ATPase (HMA) genes:**			[79,112,113,114,115,116,117]
*AtHMA2* *AtHMA4*	*Arabidopis thaliana*	Zn, Cd	
*TcHMA4*	*Thlaspi caerulescens*	Cd	
*AhHMA*	*Arabidopsis halleri*	Zn	
*OsHMA2*	*Oryza sativa*	Cd, Zn	
*HvHMA2*	*Hordeum vulgare*	Zn, Cd	
**YSL gene family:**			[112,117,118]
*AhYSL3.1* *AhYSL3.2*	*Arachis hypogaea* L.	Cu	
*AtYSL2*	*Arabidopsis thaliana*	Cu, Fe	
*OsYSL2*	*Oryza sativa*	Fe	
**NRAMP genes:**			[119,120,121,122,123]
*GmNRAMP1-7*	*Glycine max* L.	Cd, Cu, Mn, Fe	
*AtNRAMP1-* *AtNRAMP6*	*Arabidopsis thaliana*	Cd, Cu, Mn, Fe	
*MtNRAMP1*	*Medicago truncatula*	Fe	
*OsNRAMP3* *OsNRAMP5*	*Oryza sativa*	Mn, Cu	
**CDF gene family:**			[124,125,126]
*BrrMTP*		Zn, Fe, Mn	
*AtMTP*	*Arabidopsis thaliana*	Zn, Mn	
*ShMTP*	*Stylosanthes hamata*	Mn	
*OsMTP11*	*Oryza sativa*	Mn	
**CAX gene family:**			[127,128,129,130,131]
*OsCAX*	*Oryza sativa*	Mn, Cd	
*AtCAX2* *AtCAX4*	*Arabidopsis thaliana*	Cd	
**COPT gene family:**			[83,132,133,134]
*OsCOPT_1_* *OsCOPT_5_*	*Oryza sativa*	Cu	
*AtCOPT5*	*Arabidopsis thaliana*	Cu	
**ABC (ATP-binding cassett) genes:**			[135]
*OsSTAR1* *OsSTAR2*	*Oryza sativa*	Al, Cd	
*YCF1*	*Brassica juncea*	Cd	
*AtATM3*	*Arabidopsis thaliana*	Cd	
**IREG genes:**			[136,137]
*FeIREG1*	*Fagopyrum esculentum* Moench	Al	
*AtIREG1* *AtIREG3*	*Arabidopsis thaliana*	Fe, Co	

**Table A2:** Chosen of genes of heavy metals along with their tissue expression and response in plants.

Source/Target Transgenic; Plants	Genes	Main Tissue Expression	Response in Plants	References
*Brassica jouncea*/*Nicotiana tabacum*	*CAT*; *CAT3*	roots	The accumulation of the heavy metal is markedly higher in the roots than in the leaves. This lower accumulation may explain the absence of Cd^2+^ genotoxicity in leaves.	[138,139]
*Brassica oleracea*/*Arabidopsis*	*CAT1*; *CAT2*	roots	The results demonstrate that overexpression of *BoCAT1* or *BoCAT2* could reduce the phytotoxicity of H_2_O_2_ caused by high temperature in Arabidopsis. Transgenic Arabidopsis plants exhibited higher levels of RNA and *CAT*. This is the first report suggesting that *CAT*-encoding gene expression in Arabidopsis is regulated by heat stress.	[140]
*Zea mays*/*Brassica campestris*	*Cu*/*ZnSOD* and/or *CAT*	roots, shoots	Under SO_2_ stress less reduction in photosynthetic activity than wild type.	[141]
*Festuca arundinacea*	*Cu/ZnSOD* and *APX*	leaves	The mechanisms of increased antioxidative defense in transgenic tall fescue plants is the overexpression of the *CuZnSOD* and *APX* genes, which are utilized in scavenging ROS and thus provide improved tolerance to abiotic stresses.	[142]
*Triticum aestivum*/*Brasica napus*	*MnSOD*	roots, shoots	Observed 1·5- to 2·5-fold increase in total SOD activity in transgenic *B. napus* plants increased oxidative resistance compared with the wild-type plants.	[143]
*Brassica rapa*/*Escherichia coli*	*GR*; *BrGR*	-	GR participates in protection against oxidation by maintaining the adequate redox state in the intracellular environment and, thus, regulating various cellular activities Antioxidant capacity of *BrGR* protein can be examined by the induction of cell protection via the introduction of a variety of stressors, including hydrogen peroxide, menadione, or heavy metals.	[144]
*Escherichia coli*/*Nicotiana tabacum*	*DHAR/GR/GST*	leaves	Overexpression of these different enzymes enhanced salt and cold tolerance. In leaves from the *DHAR* plants, the level of *DHAR* activity increased between 1.8- and 2.7-fold when compared to the wild type. The n leaves from the *DHAR:GR* plants, this increase was found to be greater, between 3.2- and 4-fold. Leaves of the *GST* plants exhibited an increase in *GST* activity of approximately 2-fold, while this increase was greater, from 2.9- to 3.7-fold in the *GST:GR* double transformants	[145]
*Arabidopsis thaliana*/*Nicotiana tabacum*	*MDHAR/DHAR*	roots	*DHAR* but not *MDHAR* enhanced Al tolerance by maintaining the ascorbate level.	[146]
*Thlaspi caerulescens*	*ZIP*	roots, shoots	Genes are Zn regulated and Cd influx is mainly due to Zn transporters having strong preference for Zn over Cd. Zn uptake is due to overexpression of genes belonging to the *ZIP* stunted growth, chlorosis, leaf curling, and death of leaf tips.	[65,147]
*Mesembryanthemum crystallinum*	*ZIP4, IRT2*, *CAX4*, *HMA4*, *PCS1*	roots, shoots	Expression of the root genes *IRT2*, *CAX4*, *HMA4*, *PCS1*, and *ZIP4* salinity Cd stress-enhanced. For *IRT2* and *PCS1*, a cumulative effect of both stressors on gene expression was found. The salt-stressed plants subjected to a 1 mM concentration accumulated more Cd compared to the NaCl-untreated plants, and the heavy metal was stored mainly in the roots. Interestingly, this relationship was also maintained under 10 mM Cd treatment, where the salt-stressed plant roots accumulated almost 2-fold more Cd in comparison to the roots of NaCl-untreated plants. The elevated Cd amounts were deposited in shoots of NaCl-untreated and salt-stressed plants only under a 10 mM concentration.	[148]
*Mesembryanthemum crystallinum*	*IRT2*	roots	For *IRT2*, a cumulative effect of both stressors on gene expression was found. Moreover, the role of salinity stress as an upstream regulator in the halophyte *IRT2* expression scheme was suggested.	[149]
*Sedum alfredii*/*Arabidopsis thaliana*	*ZIP4*	roots, shoots	Transgenic *Arabidopsis thaliana* mutant *ZIP4-2*-expressing *SaZIP4h* reversed the Zn/Cd uptake defect, and wild-type *A. thaliana* ectopically overexpressing *SaZIP4h* displayed increased Zn accumulation both in roots and shoots. Together, these results suggest that *SaZIP4* is an important Zn uptake transporter that takes up Zn in the roots and shoots of *S. alfredii*.	[150]
*Oryza sativa*	*NRAMP1* *NRAMP5*	roots, shoots	Detoxification/sequestration of heavy metal. Revealed their role in uptake and transport of Cd Mn, and Fe. Inhibits plant growth.	[93]
*Oryza sativa*	*NRAMP1*	roots, shoots	These results suggest that *OsNRAMP1* participates in cellular Cd uptake and Cd transport within plants, and the higher expression of *OsNRAMP1* in the roots could lead to an increase in Cd accumulation in the shoots.	[151]
*Oryza sativa*	*LCT2*	roots, shoots	When grown in Cd-contaminated paddy soils, rice plants overexpressing *OsLCT2* significantly reduced Cd concentrations in the straw and grains. *OSLCT2* overexpression decreased the rate of Cd translocation from roots to shoots, and reduced Cd concentrations in xylem sap and in shoots of rice. Overexpression of *OsLCT2* reduces Cd accumulation in rice shoots and grains by limiting the amounts of Cd loaded into the xylem and restricting Cd translocation from roots to shoots of rice.	[152]
*Arabidopsis halleri*	*FDR3*	roots, shoots	Root-to-shoot translocation of heavy metals. The high expression of *FRD3 in A. halleri* contributes to metal homeostasis, but not specifically to the high accumulation of Zn in shoots of *A. halleri*.	[153]
*Lycopersicon esculentum* Mill	*GRP*	roots	*LeGRP* transcripts predominately accumulated in roots at different developmental stages, but not in leaves or ripe fruit tissues, and their levels declined gradually during plant development.	[154]
*Thlaspi arvense*	*ZNT1* *ZNT2*	roots, leaves	*T. arvense ZNT1* transcript was only detected in roots and leaves of plants grown at 0 mm Zn. Moreover, *ZNT2* is expressed only at 0 mm Zn, although it is barely detectable after hybridization. Under these conditions, and especially in roots, the expression is much lower in T. arvense than in T. caerulescens.	[155]
*Thlaspi caerulescens*	*ZNT1* *ZNT2*	roots, shoots	The expression in *T. caerulescensis* was barely Zn-responsive, suggesting that Zn hyperaccumulation might rely on a decreased Zn-induced transcriptional downregulation of these genes.	[156]
*Noccaea caerulescens*	*ZNT1*	shoots	The orthologue of the *A*. *thaliana AtZIP4* gene. Their conclusion is that *NcZNT1* plays an important role in Zn and Cd tolerance and accumulation and is involved in establishing a high metal influx into the root vasculature, important for xylem-mediated translocation of metals to the shoot.	[157]
*Medicago truncatula*	*MTP1*	vegetative organs	The expression of *MtMTP1* was detected in all vegetative organs with the highest level of expression observed in leaves.	[158]
*Arabidopsis halleri*	*MTP1*	leaves—high roots—low shoots—completely abolished	The high level of expression in leaves and completely abolished in the shoots of the plants, but weak staining, was observed in the roots.	[159]
*Mus musculus*/*Nicotiana tabacum*	*MTP1*	roots, stalk and leaves of tissues	The expression of mouse metallothionein in transplastomic plants increases mercury resistance, accumulation, and phytoremediation by the mechanism of chelation. The high level of expression in leaves of tissues.	[160]
